# How Long Should a Puerperal Woman Remain Incumbent, and What Should She Eat?

**Published:** 1865-12

**Authors:** James F. Hibberd

**Affiliations:** Richmond, Ind.


### SELECTED.

### HOW LONG SHOULD A PUERPERAL WOMAN REMAIN INCUMBENT, AND WHAT SHOULD SHE EAT?

By JAMES F. HIBBERD, M.D., Richmond, Ind.

[Read before the Wayne Co. (Ind.,) Medical Society, and after full discussion, wherein much diversity of opinion was manifested, the paper was ordered to be presented to the Cincinnati Lancet and Observer for publication.]

I have long had some doubt whether the regimen prescribed by our modern text-books for women in child-bed after ordinary uncomplicated labor, was that which is best suited to their condition, and most conducive to their early restoration to their common standard of health.

At this time, however, in fulfilling my duty as essayist, I propose to inquire into the teachings in relation to recumbency and diet only, of the puerperal state. And to the end that the drift of my remarks may be more clearly apprehended, I will state,

1st. That I believe the duration of the period of recumbency prescribed by the several obstetric authors for puerperal women is, for the most part, a mere assumption, announced as it were *ex cathedra*, and not founded on any teachings of science, nor warranted by correct observation; and

2nd. That the diet recommended by modern obstetric authors for puerperal women is unnatural and unscientific,—as a general rule injudicious, and in many instances mischievous

Let me now present what the most recent American author says on these points. I quote from Hodges' "Principles and Practice of Obstetrics," pp. 108-9: "The *diet* of the woman should always be 'low,' that is, of simple, farinaceous articles, neither hot nor cold, as the former might prove too exciting, while cold drinks might cause intestinal or uterine pain. The objections to a full diet are the predisposition to inordinate reaction, which might result not only in 'milk fever' and mammary inflammations, but also in the more severe forms of ovarian or peritoneal inflammation," etc." "The patient should be kept constantly in the recumbent *position*, not being allowed to sit up even when the bowels or bladder are emptied. The necessity for this rule is, we think, urgent, as there is danger of producing weakness or syncope from exertion, and occasionally of re-exciting uterine hæmorrhage. Moreover, the uterus being large and heavy, and the ligaments elongated, there is danger of its premature descent to the lower parts of the pelvis; and if there be any predisposition to inflammation, it will be greatly aggraved by every muscular effort. It is customary with most practitioners here and elsewhere, to allow patients to sit up after the eighth or tenth day, and soon after to commence walking. We are confident, however, that it is advisable to maintain the recumbent position almost constantly for about four weeks, and at the same time to avoid all straining efforts at stool, etc. This rule is founded on the facts already mentioned, that the uterus does not recover its natural size nor its ligaments their normal length and tonicity until four or six weeks after confinement. Hence, if premature muscular effort be made prior to this period, there is great danger of causing prolapsis, retroversion, or some other displacement of the organ, which often embitters life for many years; perhaps most women date the origin of their troubles in this respect from one of their labors. On the contrary, if they remain quiet, the natural disposition of the ligaments to contract after delivery may prevent these unfortunate accidents."

The foregoing is from the latest American author, whose book was published in 1854. Now, before indulging in any comments, and by way of comparison, let me introduce a quotation from the first American author in Obstetrics, Samuel Bard, M. D., LL. D., who published "A Compendium of the Theory and Practice of Midwifery" in 1817. He opens his fifth chapter in these words: "Among savages and half civilized nations, women made little or no change in their general conduct in consequence of child-birth, but return to their usual occupations almost immediately after delivery. Even among us, the more hardy individuals of the laboring women submit to a very short confinement; nor is it now so general among the higher classes of society, to be confined to bed for eight or ten days, and to be restricted to a particular regimen for a much longer time, as it formerly was. I believe, in so doing, they are approaching to a wiser and more natural conduct." Then, after declaring that, nevertheless, the puerperal woman requires some special attention, he says: "This attention must always be in proportion to the former habits of the woman, as well as to her general health and strength, and to the fatigue and distress she has undergone in her labor." He requires that she shall not "rise to an erect posture immediately after even the most favorable labor," and adds, "after one or two days, women should rise from their beds, and sit up for a longer or shorter time every day, according to their strength and inclination."

In regard to diet, Bard says that some practitioners "under the idea of the weakened state of their patients, have ordered a warm, cordial, stimulating diet; whilst others from apprehension of fever, have restricted them to one that is very low and abstemious. But all general rules on this subject, except that the diet should be temperate, are liable to error. In ordinary cases, common food, avoiding gross meats and spirituous liquors, may be moderately indulged in;" and "whenever it can be procured, good ripe fruit may be taken freely."

The regimen inculcated by these two men, each pre eminent in his day, is widely separated, and in strong contrast, and I am free to say that, to my mind, the fifty years progress from Bard to Hodge has been an "advance backwards." The former looks at his patients as individuals and treats each according to her previous habits, present condition, instinctive desires, and sense of ability; the latter makes a grand class of all he attends and treats them by a common rule, regardless of their former habits and present condition, totally ignoring their rational wants and instinctive desires, and heedless of their sense of ability. Unhesitatingly let us suppress the teachings of the last American, who speaks to us to-day, and in their stead revive the doctrines of the first American, whose words come to us from the depths of fifty years in the past. Hodge writes as if he were the inhabitant of a closet, and had only a distant and hypothetical view of the condition and requirements of the occupant of the lying-in chamber; while Bard talks like one standing by the bedside of his patients, giving clinical instruction from personal observation and experience. However this may be, any practical accoucheur will see at a glance that Bard is telling of things that any one may witness, and is giving instructions that can be practically carried out; while on the contrary, Hodge is portraying cases that he nor any one else has demonstrated to exist, and laying down rules that no practitioner in this longitude has sufficient influence to enforce, even if he were convinced of their propriety. Suppose a woman in firm health and good condition falls into labor, and without delay, or more than ordinary suffering, is delivered of a child. After resting two or three days, she feels able, and doubtlessly is as able as she ever will be, to get up. Now if she be a woman of good sense and vigorous will, I do not believe there is a doctor in Christendom, outside of Philadelphia, without it is Hodge, whose advice would keep her in bed for four weeks, and if there is one in that city who could, and would, keep her thus confined, it would be better for the woman if he were otherwise engaged when she sent for him.

Hodge asserts that the necessity for low diet for the puerperal woman lies in the fact that there is a "predisposition to inordinate reaction" which a full diet might develope into serious inflammation. Upon what testimony does the existence of this "predisposition to inordinate reaction" rest? There is no "inordinate reaction" in poor women who can not afford other than common food, have no special nurse, nor time to lie long in bed. Then why should the "predisposition" pertain to those who can afford these things? Before a "predisposition to inordinate reaction" can be taken as the basis of management, its existence must, in some way, be shown, simple assertion of its existence should not be sufficient for that purpose.

And, furthermore, the food must not be hot because it "might prove too exciting," nor must the patient have cold drinks lest they "might cause intestinal or uterine pain." We must suppose the author to mean by "hot" and "cold" the temperature of ordinary food and drink; and if so, I must again insist upon some evidence that they are thus mischievous. The expressed fear that they "might" excite, or cause pain, should not control rational people who reason for themselves from every day's experience.

To me it looks as if the diet had been determined upon by some intangible rule or personal whim, and the explanation of the necessity of such diet invented after the practice was established. Possibly the author had an exceptional case where a woman took a plate of hot slop made of some "simple farinaceous article," and presently became excited; or another who took a drink of cold water, followed in due time by a twinge in the bowels or a contraction of the uterus; and in a fit of abstraction, or false reasoning, he allows these two cases to dictate a rule, regardless of the two hundred cases around him, which have taken hot food and cold drinks without evil consequences, and should, by force of numbers, be allowed to establish the line of treatment.

If the modern advances in the study ot the physiology of digestion have established anything it is that the natural appetite and instinctive desire of an individual are of higher importance in determining a suitable diet for such individual than the most refined science that ignores this source of information. There is nothing in the condition of a woman in child-bed, that I am aware of, that should deprive her of the benefit of having her diet list made out, or modified, by the demands of her appetite, anb the preferences of a discriminating palate.

A woman at term goes about, doing her usual household duties, feeling well, and eating ordinary food with the others of her family. In the afternoon she feels some pain, but takes tea as usual. The pains increase, and before morning she is delivered of a child, after a normal labor. She takes a nap, and when the morning meal-time comes she does not feel like getting up, but she does feel like having some breakfast of the same kind of food she is accustomed to, but probably not so much of it. Pray what is there in her condition, present or prospective, to forbid her to have some toast, a cup of coffee, and a bit of steak with potato? Nothing, I trow, but the fact that her doctor and her nurse have long been in the habit, without rhyme or reason, of restricting the diet of women in her situation to panada, gruel, or some similar slops. Do I hear some one say that many women prefer these latter articles to the former? I venture to reply that wherever a woman manifests such a preference, it is the result of the teachings of her wrong headed attendants, and never her natural desires.

I suppose it would be a work of superrogation for me, at this day, to enter into a formal argument to show that the common mixed diet of every day life is more digestible, more easily assimilated, and generally better adapted to meet the wants of the system than the "low" diet, consisting of slops made of "simple farinaceous articles," and similar liquid preparations prescribed by our text-books for puerperal women who "are doing as well as could be expected."

Hodge is the advocate of the extremest recumbency. He complains that most practitioners allow their patients to rise in eight or ten days, while he is sure they ought to be kept down four weeks. It seems incomprehensible that a sensible Obstetrician of forty years experience should lay down such a rule as this with no more satisfactory foundation than Hodge advance. "The uterus does not recover its natural size, nor the ligaments their normal length and tonicity until four to six weeks after confinement."

Then, my dear sir, you ought, according to your own theory, to keep your puerperal women in bed for the full measure of six weeks and not stop at the modicum of four weeks. You declare four weeks to be the shortest period in which any woman's reproductive organs are restored to a normal condition after parturition, the time extending from this to six weeks, nearly all women, of course, being unrestored for more than four weeks; and yet you permit all women to abandon a recumbency at four weeks which you assert to be necessary to be maintained until full restoration. Do you not thereby become responsible for the "prolapsis, retroversion, or some other displacement of the organ" of those whose wombs, &c., do not become restored for more than four weeks? Your theory and practice do not correspond. You are inconsistent with yourself, and, therefore, in these premises, not a safe monitor.

Hodge presents us with no statistics, no facts, no experience, no arguments, to sustain his declaration that "if premature muscular efforts be made prior to this period," (i. e., four to six weeks.) "there is great danger of causing prolapsus, retroversion, or some other displacement of the organ," he only says it is so. I do not believe the position tenable.

The size of the uterus is such immediately after delivery that it is not dependent upon its ligaments to sustain it above the superior pelvic strait, its diameter is too great to permit to pass without other force than its own weight. Cazeaux says it is seven inches in diameter, and Churchill gives it "about the size of a fœtal head." No accurate measurements have been made that I am apprized of, but I presume it is within the experience of all accoucheurs that the uterus feels, through the abdominal walls, as if too large to be in any danger of passing through the strait, and as we never witness such a mishap in women whom accident or necessity has brought to their feet at such a time, we may safely conclude that the danger from this source is one of the refined phantoms of highly artificial Obstetricians.

If prolapsus could not take place, certainly we need not fear "retroversion;" and what Hodge means by "some other displacement of the organ," I do not know, but imagine he did not mean anything, except to round up a sentence.

If we were to judge of the value of a practice by the condition of those who are its subjects, there could be but little question whether the early rising from the puerperal bed were not better than long recumbency, for it is quite apparent that poor women whose necessities take them from their beds almost immediately after confinement, are much less sufferers from the ills that Hodge enumerates than the affluent and luxurious, who are able and willing, to a greater or less extent, to carry out the injunction to remain quiet and recumbent for four weeks; but the inference to be drawn from this comparison would not be just, for the former class of women, in their mode of living and exercise, are undoubtedly better prepared to stand the trials of parturition, and recuperate sooner from its disturbing influences than the latter class.

Nevertheless, if a woman is fed on slops insufficient for her nutrition when she has appetite for proper food, and is kept for weeks horizontal after she is able and willing to be up and take exercise, I believe she will be the victim of that general ill health and local disorder that Hodge prescribes these measures to prevent. In other words, the regimen prescribed for puerperal women by Hodge, if literally carried out, will, in my judgment, cause the very difficulties he is endeavoring to avoid. And this judgment rests upon the fact that those women who, in the puerperal state, are least observant of these extreme artificial rules, are the most healthy, and suffer least from child-bearing.

Moreover, it is altogether more consonant with the present state of physiological, pathological, and therapeutical knowledge that this should be the case. Simplicity and naturalness are becoming more and more the rule in all departments of medicine, why should the regimen of puerperal women be the sole exception to this better state of things? In all other branches of Obstetrics we are making advances; getting clearer views of the mechanism of labor, learning wisely to do nothing during parturition when nature is competent to do all. In this relation it has become an axiom that "meddlesome midwifery is bad;" let us carry the idea involved in this expression beyond the hour of labor and make it applicable to the post-parturient period where we appear to be becoming more and more "meddlesome" without anything to justify it.

Mr. Ferguson, in a recent clinical lecture on surgical operations (*Medical News* and *Library*, Oct., 1859,) when, speaking of after treatment, says, "some fancy that the perfection of treatment is to keep the patient low; others, that a rather stimulating course should be followed, particularly if the patient's condition is not satisfactory. For my own part, I strongly advocate giving nature much of her own way here also." And a little further again, "the less medicine the better; and experience has convinced me that the nearer a patient is kept to what may be considered his natural style of living, the less will be the shock from the operation, and the more rapid will be his recovery." No one doubts the value of this advice in surgery, and I believe the principle can be applied to midwifery with even more force than surgery.

It must not be understood that Hodge has been selected for criticism because he is more amenable to animadversion than any other modern writer on obstetrics, he, certainly, advocates a longer recumbency than any author I haved consulted, but it was not on that account his teachings were selected for the foundation of my essay, but because his book was the last one presented to us, and it is conceded on all hands to be amongst the best, if not the most excellent, work on obstetrics extant. In the matter of diet, Hodge is not nearly so precise as most recent writers; he merely requires that it be "low, simple, farinaceous articles, neither hot nor cold," while most authors name the particular articles of food to be taken, and some of them give a special diet for each day for a fortnight or more after parturition.

Perhaps we can not more profitably occupy a few minutes than in briefly reviewing the teachings of some of the modern obstetric authors on the points we have under consideration.

Bedford says "one point I wish strongly to impress upon your recollection—*keep your patient in the recumbent position for at least ten days after delivery*." Bedford must be one of the practitioners that Hodge complains of for letting their patients up in eight or ten days. In regard to diet Bedford prescribes "gruel, arrowroot, tapioca, boiled rice, tea and toast, soft boiled eggs, etc., for four or five days, and then if all "pass on favorably she may have meat and vegetables, and begin gradually to resume her ordinary diet."

Cazeaux tells us that the newly delivered takes a delightful slumber and "after the first nap she might sit up in bed for a few moments and take a little broth." Why do not the wombs of all Cazeaux's patients slip down to the bottom of the pelvis? But he requires common women to stay in bed nine days, and those in easy circumstances two weeks. He commands the woman to be limited to "a little porridge two or three times a day, and some broth during the night," for one or two days, and then return to her ordinary diet by the twelfth or fifteenth day. During the whole lying-in he allows the woman no water to drink but requires her to use instead, various preparations of art "such as a solution of gum, or an infusion of mellows, or violets or linden, the orange or chamomile flowers, etc., etc. It seems scarcely credible that a standard text-book, published only seven years ago, should promulgate such nonsense. Cazeaux is further particular to declare that no acidulated drink must even be allowed to those who nurse. Why, he does not vouchsafe to say, but I have heard women assert that acids taken by the mother will give the nursing child colic. I know of nothing in medical science to warrant such a conclusion. Did anybody ever witness such a result?

Tyler Smith directs that all patients, when circumstances will permit of it, should remain horizontal for ten days; and the diet should be light, without meat, until after lactation is established, when substantial food should be given. He warns against the protracted use of innutricious food by asserting that most cases of puerperal mania he has seen were caused by exhaustion. If any other writer has announced this conclusion I have not seen, or remembered it; but if it be true, it constitutes a very urgent reason for puerperal women being allowed an acceptable and nutritious diet.

Cock, in his excellent little manual of obstetrics requires "women to be kept in bed until after the fifth day, . . expect to be about the house in the third or fourth week." "Diet first four days, slops, tea and toast, soda biscuit, bread, panada, arrowroot, oatmeal or Indian gruel, tapioca, sago, chicken or mutton broth." Now mark the declaration that follows this farrago of fluids. "This liquid diet is not usually as digestible as solid food; after ascertaining this, use mutton, chicken, oysters, game, beef, eggs." It seems to me it would be wiser to give these latter articles in the beginning without first experimenting with the former, and the more so that no one has ever advanced a single adequate fact to deprive a puerperal woman of solid food if she prefers it. Cock closes this poragraph with the recommendation that the woman "gradually return to ordinary diet, avoiding acid articles for the sake of the child." There it is again, nothing sour to the mother lest the child suffer; but whether is the digestion of the child that is to be spoiled by the acid, or it is to make the nursling the victim of an acerbity of temper, the author does not inform us.

Meigs says: "I have found that many of my patients, and some in the class of what are called the "upper ten thousand, were destitute of all symptoms of indisposition. Such people might get up, and I have seen elegant women get up, and be about on the third day, without pretense of after indisposition." Having candidly enunciated these truths, important and full of significance as they are, it is quite surprising that a man claiming to be a philosopher should proceed thus: "still it is a safe rule to advise the keeping of the bed for many days, . . . a rest of nine days is a short rest after nine months of fatigue crowned by the exhausting conflict of labor." Nine months of fatigue! Yes, surely, given the nine months of fatigue with a conflict at the end of them, and nine days rest don't appear too much; one day per month, thirty days fatigue with a small conflict to one day's rest! There is both euphony and mathematics in this method of presenting the proposition, albeit we can discover neither science nor sense. Meig's diet is "tea, bread, gruel, vegetable jellies and panada, for three or four days."

Churchill presents the following: "The patient should never leave her bed, even to have it made, before the sixth day; and if she can be persuaded to limit her exertions to this point for eight or nine days, so much the better." "Gruel, panada, etc., for three or four days, then some broth, and on the seventh or eighth day some chicken or mutton, with some wine and water." This wine and water on the seventh or eighth day is a remnant of an old practice that stimulates the puerperal woman strongly with the alcoholic spirits, from the termination of labor, under the idea that she was very much exhausted and depressed. The practice was about extinct in the day of Churchill, but he generously pays this tribute to a former prejudice. Since Churchill, no author, I believe, teaches the use of stimulants for ordinary well doing patients but all who precede him, that I have consulted, allow wine, ale, porter, or some other form of alcohol in small quantities and dilute, sometime during the puerperal state, though all condemn it in the earlier stages.

Ramsbotham orders a change of linen in an hour or an hour and a half, but the patient must "not be allowed to get out of bed, either to sit or stand; nor must she of her own accord move hand or foot in the way of exertion." Why not at once secure her superior extremities with a straight jacket, and her inferior with gyves? What business has a woman to have a baby if she does not want to be pinioned? He permits the patient, after a week, to be laid on a sofa and have her bed made, after a fortnight she may put her feet to the "ground" and take an occasional walk about the room. He orders tea, toast, and farinaceous food for three days, some chicken or mutton broth on the fourth day, a light pudding added on the fifth; in a week allows a small quantity of solid meat, and in a fortnight "a glass of wine and water, or mild malt liquor may be taken." The delicacy of Ramsbotham is something really touching. He directs the bandage to be put on by the nurse for he "can not help thinking there is something highly indelicate in its being applied by a man." Certainly! and would it not also be best, to deliver the woman with the accoucher's hands properly gloved? Might not the possessor of such refined sensibilities receive a fatal shock through touching a woman with uncovered digits?

Ramsbotham's book was published in 1842, and we will not pursue this examination of authors farther back. We have had eight witnesses on the stand, and what a muddle their testimony makes. All agree that puerperal women, for the first day or two, should be fed on slops, or other, so called, light diet; and this is about the only point upon which they are unanimous, without it is that a puerperal woman has no natural tastes or desires that her doctor or nurse is bound to respect.

Can there be more conclusive testimony that these artificial rules are not necessary than the disagreement of the rules themselves as laid down by their several advocates. One accoucheur directs that the newly delivered woman takes a little nap, then sit up in bed and partake of some nourishment; another says she must make no exertion, move neither hand or foot for an indefinite time; a third requires her to keep in bed for four weeks at least; while most authors assert the necessity for keeping the horizontal position for eight or ten days. It is a remarkable fact that of all the women subjected to this great variety of treatment but very few perish, indeed, most of them do pretty well, everything considered. The logical inference is that the regimen prescribed in neither case is essential, but that the patient is carried through by some force not included in either prescription, and therefore better than that in either. Let us profit by this logic.

To my apprehension Bard had the warrant of both experience and science for advising that "after one or two days women should rise from their beds, and sit up for a longer or shorter time every day, according to their strength and inclination." If I were to offer an amendment it would be to strike out "one or two days," but this would be very nearly a work of supererrogation, for it would not often happen that either strength or inclination would inspire women to rise within that time. I would, however, not only allow them to sit up, but to walk about, "according to their strength and inclination," being careful to instruct them how to estimate their strength and to measure their inclination.

After uncomplicated labor, if it has been severe and protracted, the woman is tired, exhausted and sore, and needs and will unadvised, take rest of longer or shorter duration. Under this rest she will recuperate, and in a varying period, one, three, five days, she will be able to sit up, first in bed, then in a chair, and presently to move about on her feet, beginning with a very limited effect in this direction. Now, what is there in her condition that shall properly forbid her to fulfil this instinctive desire? There must be something to determine her stay in bed, what better indication to leave it can there be than her own sense of ability coupled with willingness? Do we not know that in all other cases of exhaustion, strength and vigor come more generously and pleasantly to those who leave their bed as soon as able?

Having kept the bed recumbently until more rest and a certain amount of recuperation has given a patient a sense of ability, added to a desire, to be up, is not all further confinement a source of debility that will render the patient less able to get up each succeeding day, and make her more liable to accident when she does rise? Certainly to my mind this proposition is so consonant with the present state of medical science and observation as to challenge acquiescence on all hands upon this annunciation without special proof.

But if authors are irrational and unscientific in their teachings as to the recumbency of puerperal women, what must be said of the rules and regulations laid down by them for the diet of the same patients? How odd it sounds to hear learned and experienced men give the most positive instructions for various beverages, all more or less non-satisfying to the thirst, when the woman, if left to herself, would take water, the natural, and only proper drink, fulfilling every indication and satisfying every want.

And the food: what is there in the philosophy of digestion and nutrition, or the pathology of them, for that matter, that will justify the endless particulars about slops, and panadas, and broths, and the soft end of oysters, etc. etc., even defining the day, numerically, upon which each shall be used, as if the condition of the stomach was, by some inscrutable law of the puerperal state, made fit for the food announced by a certain lapse of time, instead of our selecting the food to meet the indicated wants of the stomach and system.

To my mind, therefore, it is as clear as any other proposition in the whole round of medical science, that each puerperal patient should be looked at, and prescribed for, as an individual, having her own particular wants and necessities, and should not be regarded as one of a great number each of whom has precisely the same wants and necessities to be provided for, and satisfied, by a grand, comprehensive, common rule. And, furthermore, I verily believe that the accoucheur who does not allow the previous habits and present condition of his puerperal patients, together with their instinctive desires and reasonable wishes, to temper his judgment and modify his management, is not living up to his privileges nor his duty, no matter how exalted his wisdom otherwise, nor how profound his lore.

A difficulty of some magnitude in the management of puerperal women is encountered in the state of their own minds. They have been so long accustomed to hear that women in their situation require an especial regimen that many of them do not listen favorably to advice that does not run counter to the promptings of nature, and are driven to doubt the propriety of any regimen which is not unlike that for any other departure from common normality. I need not stop here to discuss the influence of the mental status over the physical organization for good or evil. Its power is great and acknowledged on all hands. Dr. W. F. Atlee, (*American Journal of Medical Sciences*, April, 1865, p. 468,) writes that "Dr. James Johnson eloquently says, 'The heathen philosopher (Pluto, I think,) may have carried the idea too far when he traced all diseases of the body to the mind, '*omnia corporis mala ab animo*,' but assuredly so far as my observation goes, and it has not been very limited, a great majority of corporeal disorders spring from or are aggravated by mental perturbations." For many reasons there are few states of the system where the force of these mental disturbances is more impressive than in that of women in child-bed; and those attendants will be most successful who recognize its importance and accord to it its proper weight and no more. We must be guided in giving our instructions somewhat by our patient's prejudices or preconceived opinions, but the judicious physician will always endeavor to correct, in a proper manner, whatever errors in this behalf his patients may have fallen into.

In conclusion, one may express the hope that the time will soon come when obstetric authors will give the regimen of puerperal women such considerate investigation as will enable them to present us with rules for its regulation framed in the light of more advanced medical science, and in accordance with a higher state of knowledge, abandoning the present method of treating the subject, which is, apparently, for each successive author to adopt a routine practice which shall, in some respect, differ from the routine of his predecessors. And in so doing, it appears to me, they only succeed in altering one folly by substituting or adding another folly.

